# The Complete *Amomum kravanh* Chloroplast Genome Sequence and Phylogenetic Analysis of the Commelinids

**DOI:** 10.3390/molecules22111875

**Published:** 2017-11-01

**Authors:** Mingli Wu, Qing Li, Zhigang Hu, Xiwen Li, Shilin Chen

**Affiliations:** 1 Pharmacy Faculty, Hubei University of Chinese Medicine, Wuhan 430065, Hubei, China; justuswu@163.com (M.W.); zghu0608@163.com (Z.H.); 2 Institute of Chinese Materia Medica, China Academy of Chinese Medical Sciences, Beijing 100700, China; 3 Department of Pharmacy, Changzheng Hospital, Second Military Medical University, Shanghai 200003, China; qli@smmu.edu.cn

**Keywords:** *Amomum kravanh*, chloroplast genome, phylogenetic, cardamom, commelinids

## Abstract

*Amomum kravanh* is an important edible and medicinal herb, the dried fruits of which are widely used in traditional herbal medicine as cardamom. We sequenced and analyzed the complete chloroplast (cp) genome of *A. kravanh* with herbgenomics technologies. The size of the *A. kravanh* cp genome was 162,766 bp, which consisted of long (LSC; 87,728 bp) and short (SSC; 15,390 bp) single-copy regions, separated by a pair of inverted repeats (IRs; 29,824 bp). The genome encoded 114 unique genes, including 80 protein-coding genes, 30 tRNAs and four rRNAs. A total of 299 simple sequence repeats (SSRs) were identified in the *A. kravanh* cp genome, which provides an effective method to study species identification and population genetics of the medicinal plant. Moreover, one complement, 12 forward, 12 palindrome and two reverse repeats were detected. Comparative cp genome sequence analysis of four Zingiberaceae species indicated that their intergenic spacers are highly divergent, although the gene order, gene content and genome structure differed only minimally. In particular, there was a remarkable expansion of the IR regions in the *A. kravanh* cp genome. Phylogenetic analysis strongly supported a sister relationship between *A. kravanh* and *Alpinia zerumbet*. This study identified the unique characteristics of the *A. kravanh* cp genome and might provide valuable information for future studies aiming for *Amomum* identification, and provide insights into the taxonomy of the commelinids.

## 1. Introduction

As a major plant cell organelle, the chloroplast (cp) is crucial to the growth and development of plants through its roles in photosynthesis and secondary metabolic activities. In recent years, cp genomes have been widely used for species identification, investigation of phyletic evolution and genetic engineering because of their highly conserved nature and role in monolepsis. Development of sequencing technologies has led to a sharp increase in the number of cp genome sequences; however, much remains to be achieved, particularly for medicinal species. No *Amomum* cp genome sequences have yet been reported, which has led to delays in investigation of the genetics and breeding of cardamom. In addition, the lack of complete cp genomes has hindered phylogenetic analysis of the commelinids.

*Amomum kravanh* (family Zingiberaceae) is one of the original types of cardamom plant, which is a significant traditional Chinese medicine that had been primarily imported from Thailand, Vietnam and Cambodia since the 1960s, and is now widely cultivated in southern China. Cardamom mainly contains volatile oils, which can promote the secretion of gastric juices and have antiemetic properties. Modern pharmacological research has demonstrated that the Yishen Paizhuo decoction (traditionally recognized as a decoction to support kidney function), which is mainly composed of cardamom, can be used to treat chronic renal failure (CRF) and prolong survival of patients with this condition [1]. Not only are the dried fruits of *A. kravanh* medicinal, but they can also be used as a culinary spice, similar to products from many other species in the Zingiberaceae, cassia and garlic, among others [2]. The widespread use of *A. kravanh* has increased demand for the plant, while research related to its introduction and cultivation has been insufficient, leading to the widespread occurrence of adulterants, which represent a serious health threat to consumers.

*A. kravanh* is a perennial herb of the order Zingiberales, which belongs to the clade commelinids. According to Angiosperm Phylogeny Group IV, the magnolliids, monocots and eudicots comprise the core groups of angiosperms, with commelinids the basal taxon in the monocot clade; however, there is tremendous controversy regarding the phylogeny of the commelinids, basal taxa in the order Poales. Luo et al. compared monocot pollen morphological data in an attempt to catalog these disputes [3]. Further research on the phylogenetics of the commelinids will assist in determining the evolutionary relationships among the major taxonomic categories of angiosperms.

Here we report the complete cp genome sequence of *A. kravanh*, including description of its essential characteristics and repeat sequences and comparative analyses of the cp whole genome. Moreover, we constructed a phylogenetic tree of the commelinids. These results will assist in species identification and breeding of *A. kravanh* varieties, and provide insights into the taxonomy of the commelinids. 

## 2. Results and Discussion

### 2.1. Characteristics of the A. kravanh cp Genome

**Figure 1:**
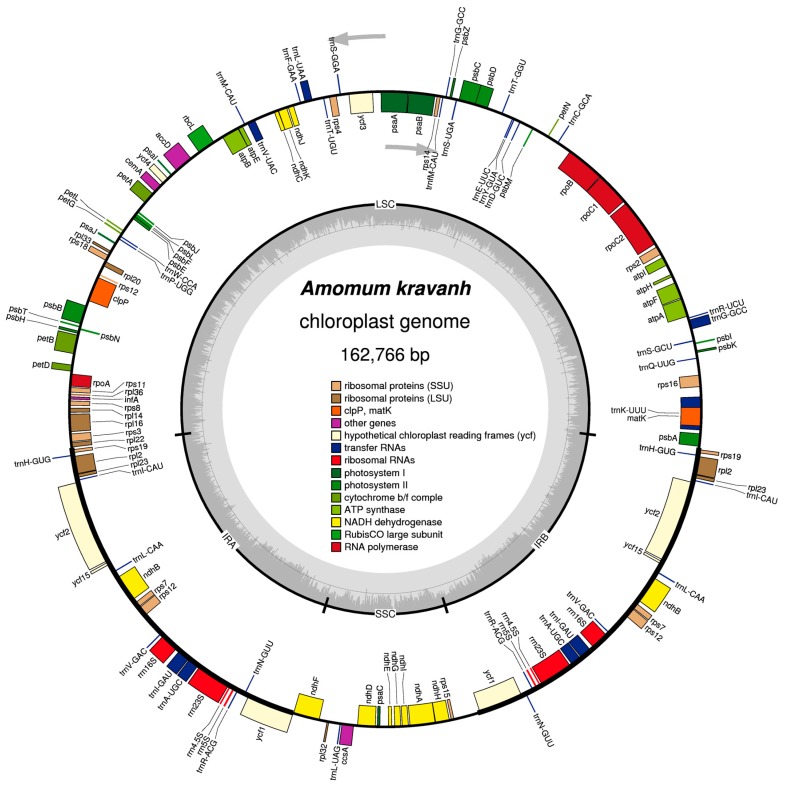
Map of the *A. kravanh* cp genome map. Genes drawn inside the circle are transcribed clockwise, and those outside are counterclockwise. Genes belonging to different functional groups are color-coded. The darker gray in the inner circle corresponds to GC content, while the lighter gray corresponds to AT content.

The complete cp genome of *A. kravanh* was revealed as 162,766 bp in size, with a typical quadripartite structure (Table 1 and Figure 1). The long (LSC; 87,728 bp) and short (SSC; 15,390 bp) single-copy regions were separated by a pair of inverted repeats (IRs; 29,824 bp each). The overall GC content of the *A. kravanh* cp genome was 31.3%, which is consistent with that reported for other cp genomes [4]. The IR regions had higher GC content than the LSC and SSC regions (Table 1). Within the protein-coding regions (CDS), the AT content of third-codon positions (70.7%) was higher than that of the first and second positions (Table 1), similar to other reported cp genomes. This bias can be used to discriminate cp DNA from nuclear and mitochondrial DNA [5,6,7,8]. In addition, the cp genome was almost equally split into coding and non-coding (introns, pseudogenes and intergenic spacer) regions.

In total, the *A. kravanh* cp genome encoded 135 functional genes, the positions of which are presented in Figure 1. There were 114 unique genes remaining after duplicates were removed, including 80 protein-coding, 30 tRNA and four rRNA genes (Table S2). Among these genes, nine protein-coding, eight tRNA and all four rRNA genes were duplicated in the IR regions. The LSC region contained 60 protein-coding and 21 tRNA genes, while 11 protein-coding genes and one tRNA gene are located in the SSC region. Introns have important roles in the regulation of gene expression, and can improve exogenous gene expression at specific locations and times; thus, introns can be useful tools for improvement of transformation efficiency [9]. Among the 114 unique genes, eight protein-coding (*rps16*, *rpl16*, *rpl2*, *rpoC1*, *ndhA*, *ndhB*, *petB* and *atpF*) and six tRNA (*trnA-UGC*, *trnG-GCC*, *trnI-GAU*, *trnK-UUU*, *trnL-UAA* and *trnV-UAC*) genes contained one intron, and three genes (*clpP*, *rps12* and *ycf3*) contained two introns. 

RSCU (relative synonymous codon usage) is a measure of non-uniform synonymous codon usage in coding sequences. RSCU values <1.00 indicate less-frequent use of a codon than expected, whereas codons used more frequently than excepted score >1.00. Based on the sequences of protein-coding genes (CDS), the codon usage frequency was estimated for the *A. kravanh* cp genome (summarized in Table 2). In total, the *A. kravanh* cp genome genes contain 27,214 codons. Other than Met, amino acid codons in the *A. kravanh* cp genome preferentially end with A or U (RSCU > 1). This codon usage pattern is similar to those reported for other cp genomes, and may be driven by a composition bias for a high proportion of A/T. In addition, codons ending in A and/or U accounted for 71.2% of all CDS codons. The majority of protein-coding genes in land-plant cp genomes employ standard ATG initiator codons. The use of the start codon (ATG) and TGG, encoding Trp, exhibited no bias (RSCU = 1) in the *A. kravanh* cp genome; however, two genes used alternatives to the AUG as a start codon as follows: *ndhD*, ATC and *rpl2*, ATA. 

### 2.2. Analysis of SSRs and Long Repeats

**Figure 2:**
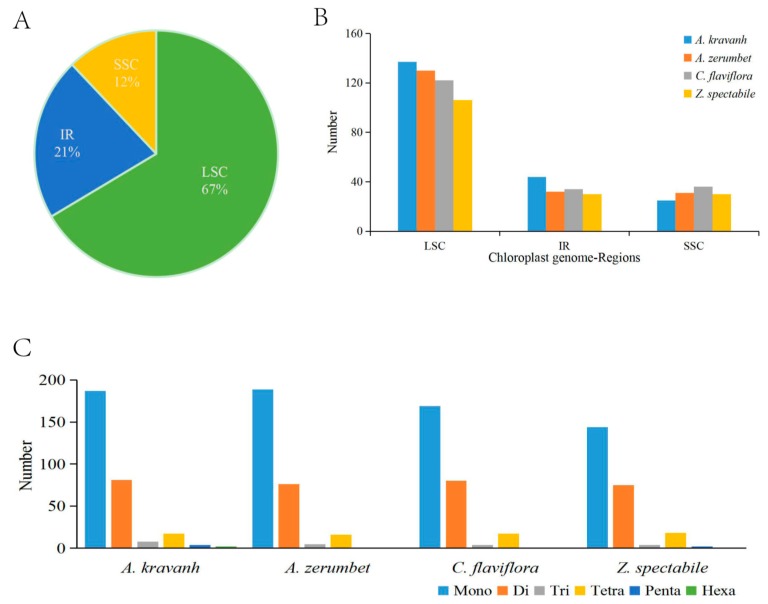
SSR (simple sequence repeats) analysis of four Zingiberaceae chloroplast genomes (*A. kravanh*, *A. zerumbet*, *C. flaviflora* and *Z. spectabile*). (**A**) Presence of SSRs in the LSC, SSC and IR regions (*A. kravanh*); (**B**) presence of SSRs in the LSC, SSC and IR regions (*A. kravanh*, *A. zerumbet*, *C. flaviflora* and *Z. spectabile*); (**C**) presence of polymers in the cp genome of *A. kravanh*, *A. zerumbet*, *C. flaviflora* and *Z. spectabile*.

The SSRs, also known as microsatellites, are a group of tandem repeated sequences, which generally consist of 1–6 nucleotide repeat units, and are widely distributed in cp genomes. SSRs are important for plant typing and are widely used as molecular markers for species identification [10,11,12]. The distribution of SSRs in the *A. kravanh* cp genome was analyzed in this study, and a total of 299 SSRs were detected, including 187 mono-, 81 di-, 8 tri-, 17 tetra-, four penta- and two hexa-nucleotide SSRs, the majority of which were located in the LSC region. Our results are consistent with the opinion that SSRs in cp genomes are generally composed of short polyadenine (polyA) or polythymine (polyT) repeats, which contribute to the A/T richness of cp genomes [13]. Furthermore, we counted the number of SSRs among four *Zingiberaceae* cp genomes (*A. kravanh*, *Alpinia zerumbet*, *Curcuma flaviflora* and *Zingiber spectabile*) and found that there was minimal difference in the distribution pattern and number of SSRs among the four cp genomes (Figure 2), although hexa-nucleotide SSRs were only identified in the *A. kravanh* cp genome. These results will undoubtedly provide cp SSR marker information for the analysis of genetic diversity in *A. kravanh* and its related species.

Repeat structure analysis revealed 27 long repeats in total: one complement, 12 forward (direct), 12 palindrome (inverted) and two reverse repeats in the *A. kravanh* cp genome (Table 3). More than half of the repeats were located in the intergenic or intronic regions, and the rest in protein-coding regions. The majority of these repeats were between 30 and 60 bp, while the *ycf1* gene possessed the longest palindrome repeats (up to 181 bp). Two pairs of repeats are associated with tRNA genes (*trnK-UUU*, *trnI-CAU*). In addition, one complement, seven forward, six palindromic and two reverse repeats were distributed in the LSC region. Short dispersed repeats are considered to be a major factor promoting cp genome rearrangements, which may facilitate intermolecular recombination and create diversity among the cp genomes in a population. Hence, the repeats identified in this study will provide valuable information to support investigation of the phylogeny of *Amomum* and *A. kravanh* population studies.

### 2.3. Comparative Chloroplast Genomic Analysis

**Figure 3:**
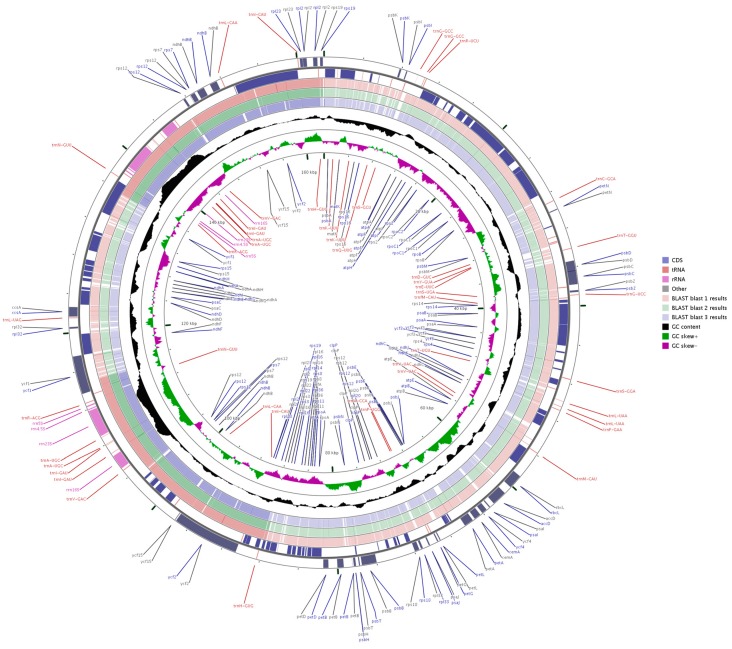
Genome comparison of three commelinid cp genomes (from blast 1 to 3: *A. zerumbet*, *C. flaviflora* and *Z. spectabile*) with that of *A. kravanh* using GCView. The two outer narrow rings show gene positions based on the *A. kravanh* cp genome. The inner two rings indicate GC skew in *A. kravanh*. GC skew+ indicates G > C, GC skew- indicates G < C.

Three sequences representing the Zingiberaceae (*A. zerumbet*, *C. flaviflora* and *Z. spectabile*) were selected for comparison with *A. kravanh*. Pairwise cp genome alignment between *A. kravanh* and the other three cp genomes revealed a high degree of synteny conservation (Figures S1–S3). The overall sequence identity among the four Zingiberaceae cp genomes was plotted using mVISTA and CGView, with the annotated *A. kravanh* sequence as the reference (Figure 3 and Figure S4). The comparison showed that the IR regions were less divergent than the LSC and SSC. In addition, the coding regions were more conserved than the non-coding regions, and the most highly divergent regions among the four cp genomes were in the intergenic spacers, including *trnS-GCU-trnG-GCC*, *atpH-atpI*, *trnC-GCA-petN*, *trnE-UUC-trnT-GGU*, *trnT-GGU-psbD*, *ndhC-trnV-UAC*, *petA-psbJ* and *psbE-petL* in the LSC, and *ndhF-rpl32*, *rpl32-trnL-UAG*, *psaC-ndhE* and *rps15-ycf1* in SSC, similar to other plant cp genomes [14]. Moreover, the most divergent coding regions are the *ndhA*, *ycf1*, *petB* and *ycf2* genes in the cp genomes. Sequence identity analysis showed that *A. zerumbet* had the highest sequence similarity to *A. kravanh*, which was consistent with the results of phylogenetic analysis. In our study, we observed that all eight rRNA genes were the most conserved. The highly divergent regions identified in this research could be used to develop markers or specific barcodes that would maximize the ability to differentiate species within the Zingiberaceae.

### 2.4. IR Contraction and Expansion

**Figure 4:**
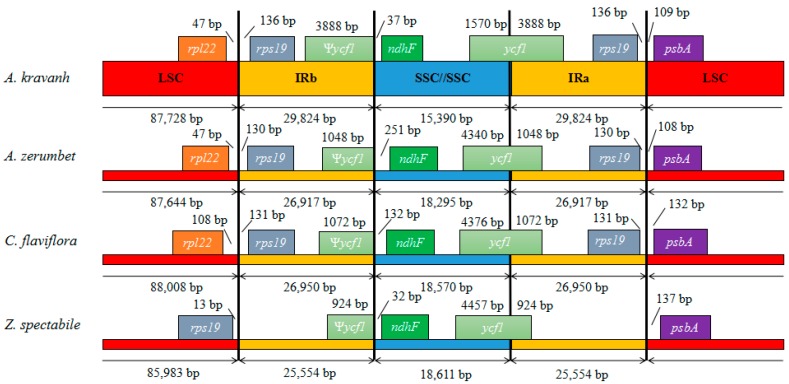
Comparison of the borders of LSC, SSC and IR regions among four chloroplast genomes. Ψ, pseudogenes.

Contraction and expansion at the borders of IR regions are common evolutionary events considered the main reason for size differences among cp genomes [11,13,14,15,16]. Detailed comparison of the junctions between the IR and LSC/SSC regions among the four Zingiberaceae cp genomes (*A. kravanh*, *A. zerumbet*, *C. flaviflora* and *Z. spectabile*) is presented in Figure 4. In addition, a comparison of cp genome size among the examined Zingiberaceae species is provided in Table S3. As for other reported cp genomes, the IRa/SSC border was generally positioned in the coding region of the *ycf1* gene, resulting in a deletion of the 5′ end of the gene, to generate a pseudogene at the IRb/SSC border. The *ycf1* pseudogene has proven useful in analysis of cp genome variation in higher plants and algae [17], and varies in length from 924–3888 bp in the cp genomes compared. In the *A. kravanh* cp genome in particular, there was a remarkable expansion of the IR regions, resulting in the largest IR (29,824 bp) and smallest SSC (15,390 bp) regions relative to the other three cp genomes (Table S3). The photosynthetic gene, *ndhF*, was 37, 251, 132 and 32 bp from the IRb/SSC border in *A. kravanh*, *A. zerumbet*, *C. flaviflora* and *Z. spectabile*, respectively. The *rps19* gene, which is one of the most abundant transcripts in the cp genome, was situated in the IR regions of all the cp genomes included in the comparison, except for *Z. spectabile*. In the *Z. spectabile* cp genome, *rps19* was located in the LSC region, which may be because of the contraction of the IR regions, which makes the whole cp genome of this species shorter. Although the IR/LSC boundaries are not static among the Zingiberaceae species cp genomes analyzed, the dynamic processes appear to have been confined to conservative expansions and contractions, similar to other eukaryon plants [18].

### 2.5. Analysis of Synonymous and Non-Synonymous Substitution Rates 

**Figure 5:**
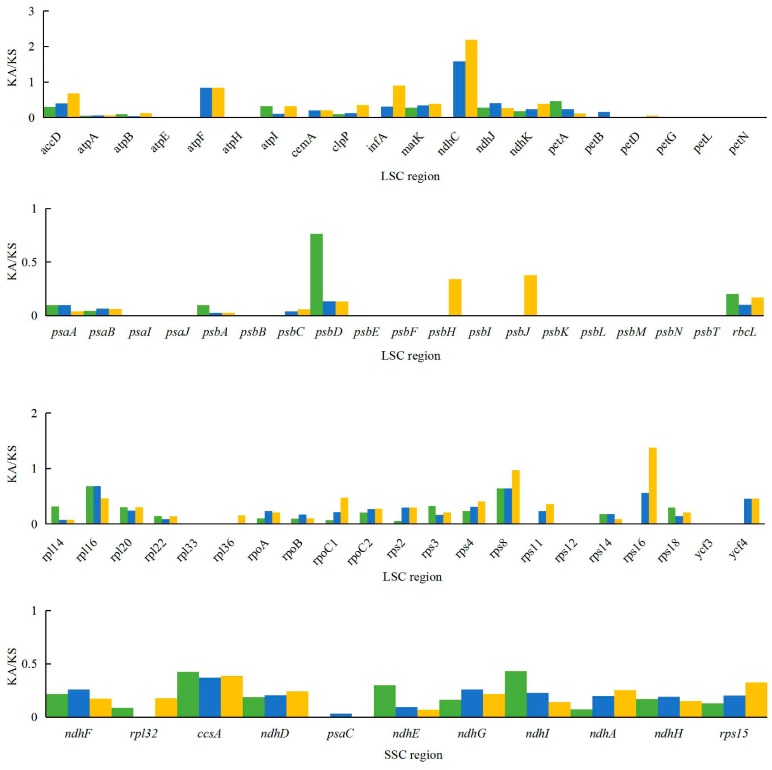

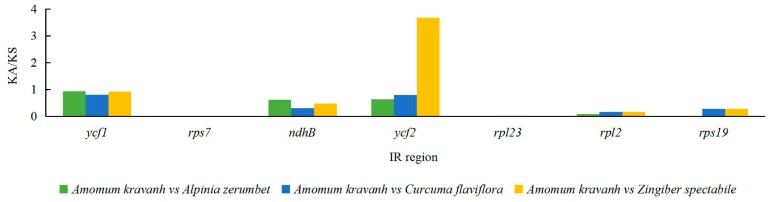
Ka/Ks values of 80 protein-coding genes of *A. kravanh*, *A. zerumbet*, *C. flaviflora* and *Z. spectabile*.

The Ka/Ks ratio is widely used to evaluate the evolutionary forces on specific groups of genes; ratios > 1 indicate positive selection, values < 1 indicate negative (purifying) selection, and values of 1 indicate neutral selection. In this study, a total of 80 protein-coding genes in the *A. kravanh* cp genome were included in the analysis of synonymous and non-synonymous substitutions rates, relative to *A. zerumbet*, *C. flaviflora* and *Z. spectabile* (Figure 5). The Ka/Ks ratios of the majority of genes were <1 compared with the three closely related species. This indicates that the majority of protein-coding regions in the *A. kravanh* cp genome have been under strong purifying selection during evolution. In addition to genes with high Ka/Ks values, the Ks values (≥1) of *ndhC*, *rps16* and *ycf2* indicate they have undergone positive selection, which was also found from analysis of other cp genomes [19]. However, compared with previous studies, it is clear that cp genes may be influenced by varying levels of selection pressure in different plants.

### 2.6. Phylogenetic Analysis 

**Figure 6:**
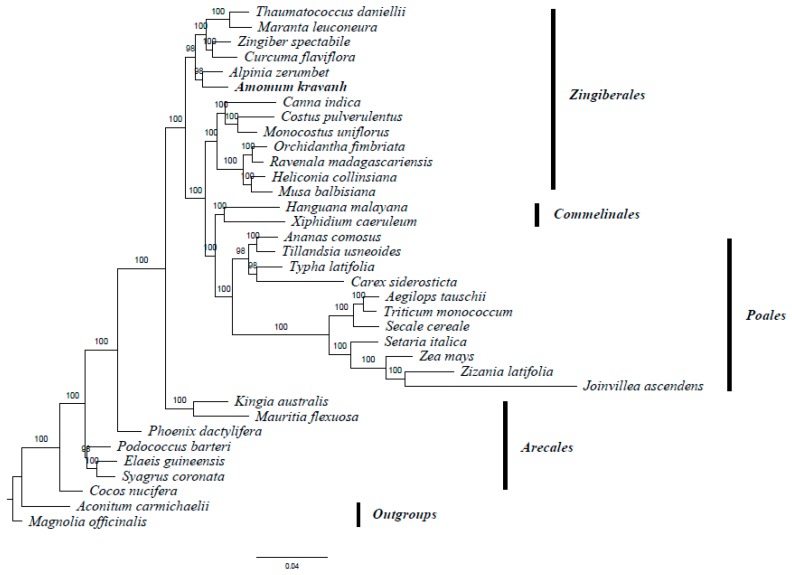
ML phylogenetic tree of 33 taxa in the commelinid clade based on the concatenated sequences of 58 shared chloroplast protein-coding genes. Numbers above each node are ML bootstrap values >50%. *Magnolia officinalis* and *Aconitum carmichaelii* were used as outgroups.

*A. kravanh* belongs to the Zingiberales order in the commelinid clade. Several studies have reported analysis of phylogenetic relationships within the commelinid clade based on morphological and molecular characteristics [20,21,22,23]. The availability of complete cp genome sequences of *A. kravanh* and other commelinid species provided us with data to investigate the molecular evolution of *A. kravanh* and the phylogeny of the commelinid clade. In this study, we performed multiple sequence alignments of 58 protein-coding genes commonly present in cp genomes from species belonging to 33 commelinid-clade species. The cp genomes of *Magnolia officinalis* and *Aconitum carmichaelii* were included as outgroups. On the basis of the GTR + G + I nucleotide substitution model recommended, a total of 39,750 nucleotide positions were analyzed, and the best-scoring ML (maximum likelihood) tree (final ML optimization likelihood = −281,929.050009) is presented in Figure 6. MP (maximum parsimony) analysis was also performed (Figure S5), and the resulting topology was relatively consistent with that produced by ML analysis, with a consistency index (CI) of 0.5453, a homoplasy index (HI) of 0.4547 and a retention index (RI) of 0.6772. The relationships among major basal commelinid lineages in our analyses are basically consistent with APG IV. ML and MP phylogenetic results both strongly supported the position in the Zingiberaceae of *A. kravanh* as a sister of the closely related species *A. zerumbet*, which was also used as cardamom in ancient China [24]. The results of this study should be validated in future analyses with more complete cp genomes and sophisticated analytical approaches.

## 3. Materials and Methods

### 3.1. DNA Sequencing and Genome Assembly 

Fresh *A. kravanh* leaves were collected from cultivated fields in Hainan Province, China. Total chloroplast DNA (cpDNA) was extracted from approximately 100 g of leaves using the sucrose gradient centrifugation method, as improved by Li et al. (2012) [25]. The concentration of cpDNA was estimated by measuring A260 and A280 using an ND-2000 spectrometer (Nanodrop Technologies, Wilmington, DE, USA); samples were also visually examined by gel electrophoresis. Pure cpDNA was used to construct shotgun libraries, which were sequenced by Herbgenomics Technologies on the 454 GS FLX Titanium platform (Roche Diagnostics, Basel, Switzerland), according to the manufacturer’s instructions. The resulting sff-file was pre-processed by trimming low-quality (Q < 20) and short (L < 50 bp) reads. This resulted in about 60× coverage of this cp genome. Cleaned reads were used for sequence assembly using GS FLX De Novo Assembler Software (Newbler V2.6). To verify the assembly, the four junctions between the inverted repeat (IR) and long single-copy/short single-copy (LSC/SSC) regions were confirmed by PCR amplification and Sanger sequencing.

### 3.2. Genome Annotation

The initial gene annotation of the *A. kravanh* cp genome was performed using CPGAVAS (http://www.herbalgenomics.org/cpgavas) [26] and confirmed with BLAST and DOGMA (http://dogma.ccbb.utexas.edu/) [27]. tRNA genes were identified using tRNAscanSE (http://lowelab.ucsc.edu/tRNAscan-SE/) [28] with default settings. Then, the position of each gene was manually corrected using Apollo [29], according to the positions of start and stop codons. The circular cp genome map was drawn using OGDRAW (http://ogdraw.mpimp-golm.mpg.de/) [30]. The final cp genome of *A. kravanh* was submitted to GenBank (Accession Number: MF991963).

### 3.3. Sequence Analyses

To analyze the characteristics of synonymous codon usage, relative synonymous codon usage values (RSCU), codon usage and GC content were determined using MEGA7 [31]. Simple sequence repeats (SSRs) were detected with MISA [32], using the following thresholds: 8, 4, 4, 3, 3 and 3 repeat units for mono-, di-, tri-, tetra-, penta- and hexa-nucleotide SSRs, respectively. We performed the same SSR analysis for three other Zingiberaceae cp genomes, and conducted analysis with mVISTA to assess the relative number of SSRs in the four different cp genomes. For analysis of repeat structures, REPuter (http://bibiserv.tech--fak.uni-bielefeld.de/reputer/) [33] was used to visualize forward, reverse, palindrome and complement sequences of size ≥30 bp and identity ≥90% in the cp genome. All repeats recognized were manually verified, and redundant results removed.

### 3.4. Genome Comparison

MUMmer [34] was used to perform pairwise cp genomic alignment, and dotplots were drawn using a Perl script. The mVISTA program was employed in Shuffle-LAGAN mode [35,36] to compare the cp genome of *A. kravanh* with those of *Alpinia zerumbet* (JX088668), *Curcuma flaviflora* (KR967361) and *Zingiber spectabile* (JX088661). Additionally, the *A. kravanh* cp genome was compared with three available commelinid cp genomes (*A. zerumbet*, JX088668; *Musa balbisiana*, KT595228; *Kingia australis*, JX051651) using CGView (http://stothard.afns.ualberta.ca/cgview_server/index.html) [37]. GC distributions were measured based on GC skew, using the following equation: GC skew = (G − C)/(G + C). 

### 3.5. Analysis of Synonymous and Non-Synonymous Substitution Rates 

The *A. kravanh* cp genome sequence was compared with those of *A. zerumbet*, *C. flaviflora* and *Z. spectabile*. To evaluate synonymous (Ks) and non-synonymous (Ka) substitution ratios, shared individual protein-coding exons were extracted using a Python script and separately aligned with MEGA7. Ks and Ka substitution rates for each protein-coding gene were estimated in DnaSP [38]. 

### 3.6. Phylogenetic Analysis

For phylogenetic analysis, 33 complete cp genome sequences were downloaded from NCBI (Table S1), and a dataset of 58 protein-coding genes commonly present in the 35 cp genomes included in the analysis was used to construct the phylogenetic tree. All shared genes were aligned separately using ClustalW2 [39]. Jmodeltest [40] was employed to determine the most appropriate model for maximum likelihood (ML), based on Akaike information criterion (AIC), then ML analysis was performed using the tool RAxML-HPC 2.7.6.3 [41] in XSEDE at the CIPRES Science Gateway (http://www.phylo.org/) [42] with 1000 bootstrap replicates. Similarly, maximum parsimony (MP) analysis was performed using PAUP in XSEDE. *Magnolia officinalis* and *Aconitum carmichaelii* were included as outgroups.

## 4. Conclusions

The complete cp genome sequence of *A. kravanh* was assembled, annotated and analyzed in this study. Detailed comparisons with the cp genomes of three other Zingiberaceae species demonstrated that the *A. kravanh* cp genome has clearly undergone expansion of the IR regions, while the gene content, gene order, genome structure and SSRs were broadly similar. Repeated sequences, together with the aforementioned SSRs, are informative sources for the development of new molecular markers. Phylogenetic relationships among 33 commelinid species strongly supported a sister relationship between *A. kravanh* and *A. zerumbet*. The comprehensive data presented in this study provide insights into the characteristics of the entire *A. kravanh* cp genome and the phylogenetic relationships within the commelinid clade, which will facilitate a breeding program, genetic engineering and evolutionary studies among the major angiosperm taxonomic categories. 

## Figures and Tables

**Table 1:** Base composition in the *Amomum kravanh* chloroplast genome.

		T (U) (%)	C (%)	A (%)	G (%)	Length (bp)
LSC		33.8	17.3	32.3	16.6	87,728
SSC		34.2	15.6	36.0	14.2	15,390
IRA		28.8	19.8	30.1	21.3	29,824
IRB		30.1	21.3	28.8	19.8	29,824
Total		32.2	18.3	31.6	17.8	162,766
CDS		31.6	17.2	31.3	19.9	81,642
	1st position	24.0	18.3	31.2	26.6	27,214
	2nd position	32.0	20.2	29.9	17.5	27,214
	3rd position	38.0	13.2	32.8	15.5	27,214

LSC, large single-copy region; SSC, small single-copy region; IR, inverted repeat sequence; CDS, protein-coding regions.

**Table 2:** Codon–anticodon recognition patterns and codon usage in the *A. kravanh* chloroplast genome.

Amino Acid	Codon	Count	RSCU	tRNA	Amino Acid	Codon	Count	RSCU	tRNA
Phe	UUU	997	1.29		Tyr	UAU	818	1.58	
Phe	UUC	545	0.71	*trnF-GAA*	Tyr	UAC	219	0.42	*trnY-GUA*
Leu	UUA	900	1.93	*trnL-UAA*	Stop	UAA	49	1.69	
Leu	UUG	579	1.24	*trnL-CAA*	Stop	UAG	21	0.72	
Leu	CUU	586	1.25		His	CAU	526	1.6	
Leu	CUC	192	0.41		His	CAC	132	0.4	*trnH-GUG*
Leu	CUA	389	0.83	*trnL-UAG*	Gln	CAA	725	1.54	*trnQ-UUG*
Leu	CUG	159	0.34		Gln	CAG	218	0.46	
Ile	AUU	1168	1.47		Asn	AAU	1010	1.54	
Ile	AUC	438	0.55	*trnI-GAU*	Asn	AAC	298	0.46	*trnN-GUU*
Ile	AUA	772	0.97	*trnI-CAU*	Lys	AAA	1105	1.47	*trnK-UUU*
Met	AUG	636	1	*trn(f)M-CAU*	Lys	AAG	399	0.53	
Val	GUU	535	1.45		Asp	GAU	896	1.64	
Val	GUC	168	0.46	*trnV-GAC*	Asp	GAC	196	0.36	*trnD-GUC*
Val	GUA	571	1.55	*trnV-UAC*	Glu	GAA	1144	1.51	*trnE-UUC*
Val	GUG	200	0.54		Glu	GAG	371	0.49	
Ser	UCU	622	1.74		Cys	UGU	240	1.55	
Ser	UCC	352	0.99	*trnS-GGA*	Cys	UGC	69	0.45	*trnC-GCA*
Ser	UCA	427	1.2	*trnS-UGA*	Stop	UGA	17	0.59	
Ser	UCG	196	0.55		Trp	UGG	469	1	*trnW-CCA*
Pro	CCU	454	1.64		Arg	CGU	372	1.35	*trnR-ACG*
Pro	CCC	206	0.74		Arg	CGC	89	0.32	
Pro	CCA	328	1.18	*trnP-UGG*	Arg	CGA	353	1.28	
Pro	CCG	122	0.44		Arg	CGG	120	0.43	
Thr	ACU	546	1.57		Arg	AGA	545	1.97	*trnR-UCU*
Thr	ACC	241	0.69	*trnT-GGU*	Arg	AGG	178	0.64	
Thr	ACA	441	1.27	*trnT-UGU*	Ser	AGU	436	1.22	
Thr	ACG	161	0.46		Ser	AGC	108	0.3	*trnS-GCU*
Ala	GCU	631	1.82		Gly	GGU	614	1.39	
Ala	GCC	202	0.58		Gly	GGC	144	0.33	*trnG-GCC*
Ala	GCA	440	1.27	*trnA-UGC*	Gly	GGA	729	1.65	
Ala	GCG	115	0.33		Gly	GGG	285	0.64	

RSCU, relative synonymous codon usage.

**Table 3:** Long repeat sequences in the *A. kravanh* chloroplast genome.

No.	Size (bp)	Type	Repeat 1 Start	Repeat 1 Location	Repeat 2 Start	Repeat 2 Location	Region	E-Value
1	30	C	9027	IGS	53,756	IGS	LSC	7.08 × 10^−4^
2	30	F	10,500	*trnG-GCC*	38,870	*trnG-UCC*	LSC	7.08 × 10^−4^
3	30	F	32,612	IGS	32,641	IGS	LSC	7.08 × 10^−4^
4	30	F	33,321	IGS	133,080	IGS	LSC; IRa	7.08 × 10^−4^
5	58	F	41,076	*psaB* (CDS)	43,300	*psaA* (CDS)	LSC	7.47 × 10^−20^
6	37	F	41,120	*psaB* (CDS)	43,344	*psaA* (CDS)	LSC	2.36 × 10^−9^
7	30	F	70,833	IGS	70,860	IGS	LSC	6.46 × 10^−9^
8	42	F	71,482	*rps18* (CDS)	71,503	*rps18* (CDS)	LSC	1.19 × 10^−10^
9	46	F	90,440	*trnI-CAU*	90,490	IGS	IRb	2.08 × 10^−16^
10	30	F	93,108	*ycf2* (CDS)	93,129	*ycf2* (CDS)	IRb	7.08 × 10^−4^
11	30	F	157,337	*ycf2* (CDS)	157,358	*ycf2* (CDS)	IRa	7.08 × 10^−4^
12	46	F	159,958	IGS	160,008	IGS	IRa	2.08 × 10^−16^
13	30	F	159,977	IGS	160,027	IGS	IRa	7.08 × 10^−4^
14	34	P	3990	*trnK-UUU*	3996	*trnK-UUU*	LSC	4.08 × 10^−6^
15	31	P	8746	IGS	47,581	*trnS-GGA*	LSC	1.96 × 10^−4^
16	32	P	30,943	IGS	30,973	IGS	LSC	5.41 × 10^−5^
17	30	P	33,321	IGS	117,383	*ycf1* (CDS)	LSC; IRb	7.08 × 10^−4^
18	30	P	34,662	IGS	62,566	IGS	LSC	7.08 × 10^−4^
19	32	P	39,185	IGS	39,226	IGS	LSC	4.04 × 10^−10^
20	31	P	67,014	IGS	67,069	IGS	LSC	6.76 × 10^−6^
21	46	P	90,440	*trnI-CAU*	159,958	IGS	IRb; IRa	2.08 × 10^−16^
22	46	P	90,490	IGS	160,008	IGS	IRb; IRa	2.08 × 10^−16^
23	30	P	93,108	*ycf2* (CDS)	157,335	*ycf2* (CDS)	IRb; IRa	7.08 × 10^−4^
24	30	P	93,129	*ycf2* (CDS)	157,356	*ycf2* (CDS)	IRb; IRa	7.08 × 10^−4^
25	181	P	117,370	*ycf1* (CDS)	132,942	*ycf1* (CDS)	IRb; IRa	7.93 × 10^−100^
26	31	R	13,116	*atpF* (intron)	34,886	IGS	LSC	6.76 × 10^−6^
27	34	R	33,365	IGS	33,374	IGS	LSC	4.08 × 10^−6^

C, complement; F, forward; P, palindrome; R, reverse; IGS, intergenic spacer region.

## Supplementary Materials

Supplementary materials are available online.
