# Supplementary material for: The Complete Amomum kravanh Chloroplast Genome Sequence and Phylogenetic Analysis of the Commelinids
Source: Molecules. 2017 Nov 1;22(11):1875. doi: 10.3390/molecules22111875 (PMC6150383; doi:10.3390/molecules22111875)
Supplement: Supplementary file 1 [file molecules-22-01875-s001.pdf]

Supplementary Material

Table S1. The list of accession numbers of the chloroplast genome sequences used in the phylogenetic analysis.

No.	Taxon	Family	Order	GenBank Accession Number
1	*Ananas comosus*	Bromeliaceae	Poales	AP014632
2	*Elaeis guineensis*	Arecaceae	Arecales	JF274081
3	*Kingia australis*	Dasypogonaceae	Dasypogonales	JX051651
4	*Heliconia collinsiana*	Heliconiaceae	Zingiberales	JX088660
5	*Zingiber spectabile*	Zingiberaceae	Zingiberales	JX088661
6	*Alpinia zerumbet*	Zingiberaceae	Zingiberales	JX088668
7	*Xiphidium caeruleum*	Haemodoraceae	Commelinales	JX088669
8	*Ravenala madagascariensis*	Strelitziaceae	Zingiberales	KF601568
9	*Orchidantha fimbriata*	Lowiaceae	Zingiberales	KF601569
10	*Canna indica*	Cannaceae	Zingiberales	KF601570
11	*Maranta leuconeura*	Marantaceae	Zingiberales	KF601571
12	*Monocostus uniflorus*	Costaceae	Zingiberales	KF601572
13	*Costus pulverulentus*	Costaceae	Zingiberales	KF601573
14	*Thaumatococcus daniellii*	Marantaceae	Zingiberales	KF601575
15	*Setaria italica*	Poaceae	Poales	KJ001642
16	*Zizania latifolia*	Poaceae	Poales	KM282190
17	*Carex siderosticta*	Cyperaceae	Poales	KP751906
18	*Podococcus barteri*	Arecaceae	Arecales	KR347117
19	*Mauritia flexuosa*	Arecaceae	Arecales	KT312914
20	*Hanguana malayana*	Hanguanaceae	Commelinales	KT312930

21	*Cocos nucifera*	Arecaceae	Arecales	KX028884
22	*Joinvillea ascendens*	Joinvilleaceae	Poales	KX035098
23	*Tillandsia usneoides*	Bromeliaceae	Poales	KY293680
24	*Zea mays*	Poaceae	Poales	X86563
25	*Typha latifolia*	Typhaceae	Poales	GU195652
26	*Phoenix dactylifera*	Arecaceae	Arecales	GU811709
27	*Magnolia officinalis*	Magnoliaceae	Ranunculales	JN867579
28	*Triticum monococcum*	Poaceae	Poales	KC912690
29	*Secale cereale*	Poaceae	Poales	KC912691
30	*Aegilops tauschii*	Poaceae	Poales	JQ754651
31	*Musa balbisiana*	Musaceae	Zingiberales	KT595228
32	*Curcuma flaviflora*	Zingiberaceae	Zingiberales	KR967361
33	*Syagrus coronata*	Arecaceae	Arecales	KT896548
34	*Aconitum carmichaelii*	Ranunculaceae	Ranales	KY407560

Table S2. Genes in the *A. kravanh* chloroplast genome

**Gene category**	**Gene group**	**Gene names**
Self-replication	rRNA	*rrn16*[a], *rrn23*[a], *rrn5*[a], *rrn4.5*[a]
	tRNA	*trnA-UGC*[*,a], *trnC-GCA, trnD-GUC, trnE-UUC, trnF-GAA, trnG-GCC*[*], *trnG-UCC, trnH-GUG*[a], *trnI-CAU*[a], *trnI-GAU*[*,a], *trnK-UUU*[*], *trnL-UAA*[*], *trnL-UAG, trnL-CAA*[a], *trnM-CAU, trnfM-CAU, trnN-GUU*[a], *trnP-UGG, trnQ-UUG, trnR-ACG*[a], *trnR-UCU, trnS-GCU, trnS-UGA, trnS-GGA, trnT-GGU, trnT-UGU, trnV-UAC*[*], *trnV-GAC*[a], *trnW-CCA, trnY-GUA*
	Small subunit of ribosome	*rps2, rps3, rps4, rps7*[a], *rps8, rps11, rps12*[**,a], *rps14, rps15,*

	Large subunit of ribosome	*rps16*, rps18, rps19*[a]
		rpl14, rpl16, rpl2**[,a]*, rpl20, rpl22, rpl23*[a]*, rpl32, rpl33, rpl36*
	DNA dependent RNA polymerase	
		rpoB, rpoC1, rpoC2, rpoA*
	Translational initiation factor	
		infA
Photosynthetic	Subunits of NADH-dehydrogenase	*ndhA*, ndhB**[,a]*, ndhC, ndhD, ndhE, ndhF, ndhG, ndhH, ndhI, ndhJ, ndhK*
		*psbA, psaB, psaC, psaI, psaJ, ycf3**, ycf4*
	Subunits of photosystem I	*psbA, psbB, psbC, psbD, psbE, psbF, psbH, psbI, psbJ, psbK, psbL, psbM, psbN, psbT, psbZ*
	Subunits of photosystem II	
		petN, petA, petL, petG, petB, petD*
	Subunits of cytochrome b/f complex	*atpI, atpH, atpF*, atpA, atpE, atpB*
	Subunits of ATP synthase	*rbcL*
	Large subunit of rubisco	
Other	Maturase	*matK*
	Protease	*clpP***
	Envelope membrane protein	*cemA*
	Subunit of Acetyl-CoA-carboxylase	*accD*
		ccsA
	C-type cytochrome synthesis	
Genes of unknown function	Open Reading Frames (ORF, ycf)	*ycf1, ycf15*[a]*, ycf2*[a]
	Pseudogenes	*ycf1*

**Genes with one intron*; ***Genes with two introns*; [a]*Genes with two copies.*

Table S3. Comparison of the sizes of the chloroplast genomes of *A. kravanh* and three other Zingiberaceae species

Species	Length (bp)			
	Total genome	LSC	SSC	IR
Amomum kravanh	162,766	87,728	15,390	29,824
Alpinia zerumbet	159,773	87,644	18,295	26,917
Curcuma flaviflora	160,478	88,008	18,570	26,950
Zingiber spectabile	155,702	85,983	18,611	25,554

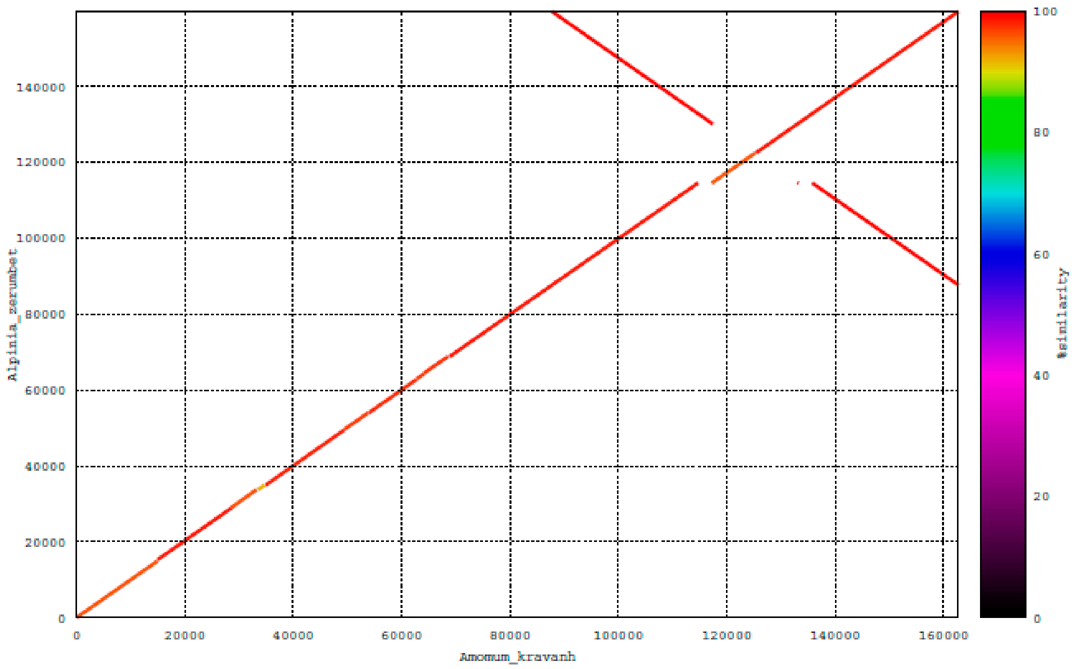

Figure S1. Alignment of the chloroplast genome sequences of *A. kravanh* and *Alpinia zerumbet*.

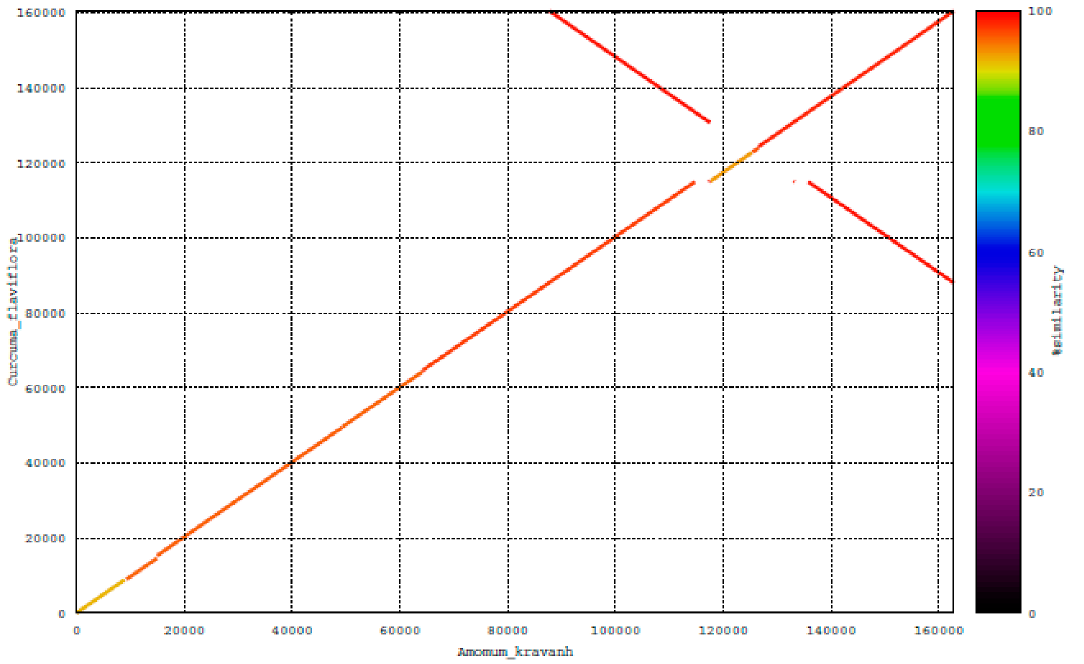

Figure S2. Alignment of the chloroplast genomes of alignment *A. kravanh* and *Curcuma flaviflora*.

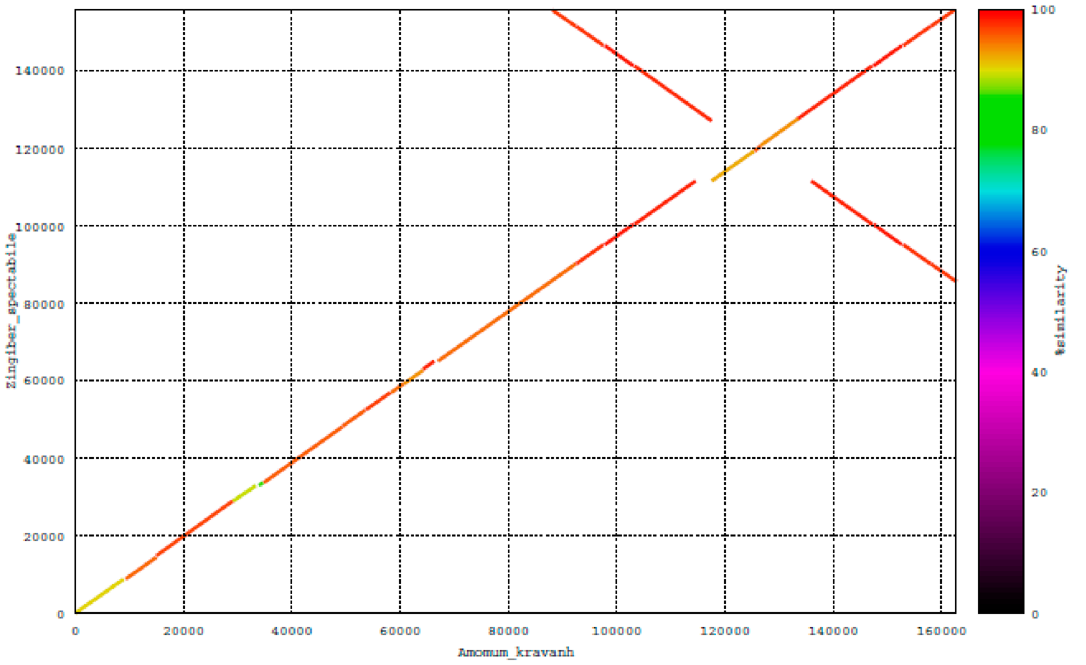

Figure S3. Alignment of the chloroplast genomes of *A. kravanh* and *Zingiber spectabile*.

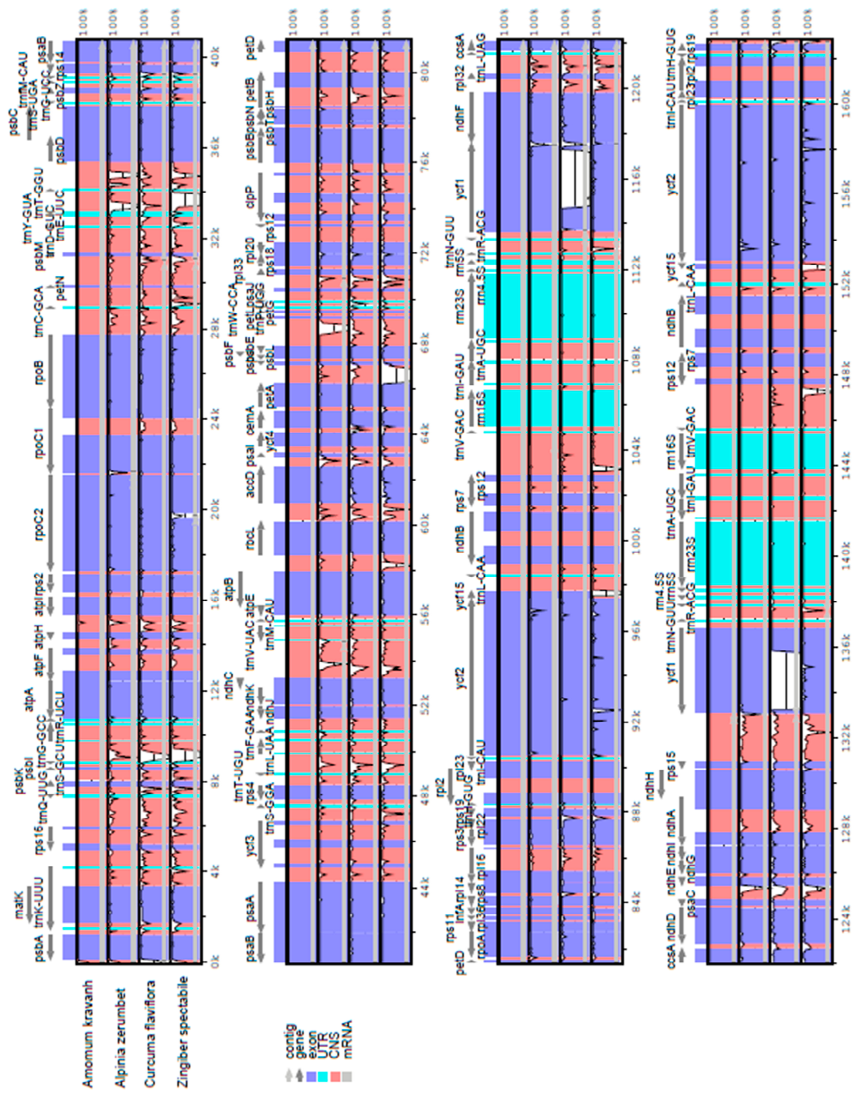

Figure S4. Comparison of four Zingiberaceae chloroplast genomes using the mVISTA program. Grey arrows and thick black lines above the alignment indicate the orientation of genes. Purple, blue, and pink bars represent exons, UTRs, and non-coding sequences (CNS), respectively. Percentage identity (50%–100%) is shown on the Y-axis. Genome regions are color-coded to indicate protein-coding exons, rRNAs, tRNAs, and conserved noncoding sequences (CNS).

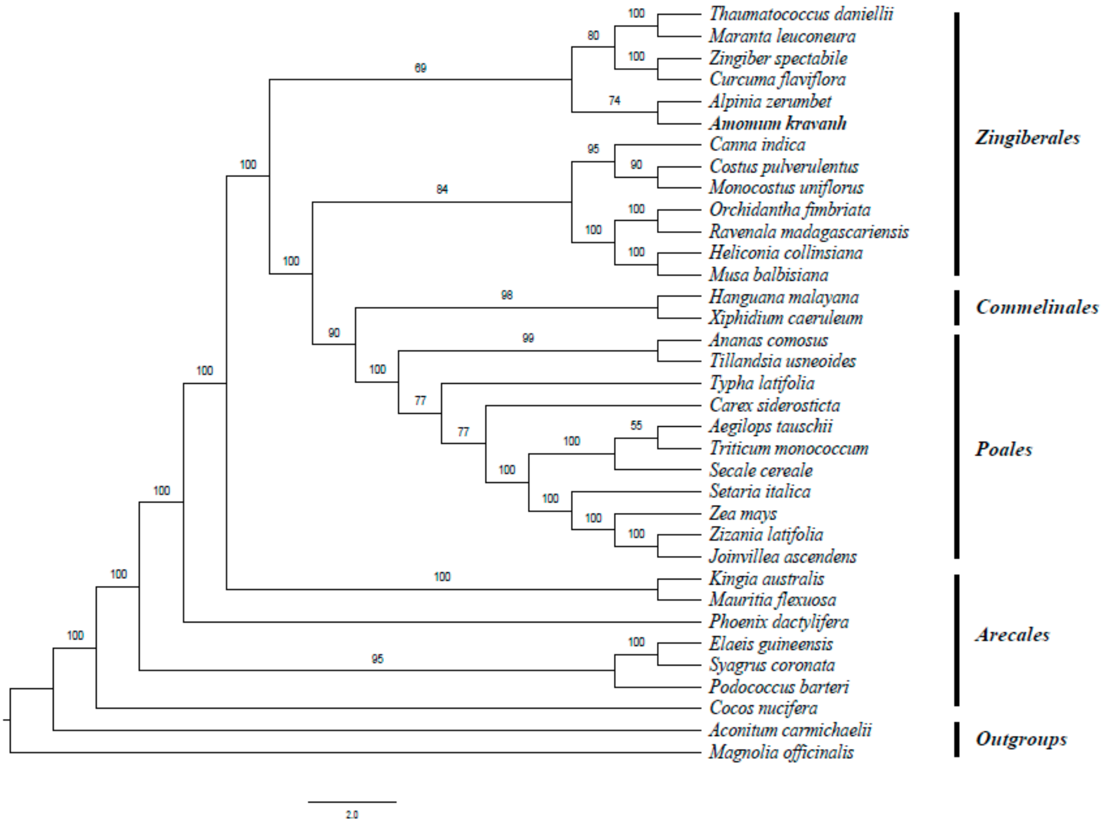

Figure S5. MP phylogenetic tree of 33 taxa in the commelinids clade based on the concatenated sequences of 58 shared chloroplast protein-coding genes. Numbers above each node are MP bootstrap values > 50%. *Magnolia officinalis* and *Aconitum carmichaelii* was used as outgroups.
